# Neutrophils: Between Host Defence, Immune Modulation, and Tissue Injury

**DOI:** 10.1371/journal.ppat.1004651

**Published:** 2015-03-12

**Authors:** Philipp Kruger, Mona Saffarzadeh, Alexander N. R. Weber, Nikolaus Rieber, Markus Radsak, Horst von Bernuth, Charaf Benarafa, Dirk Roos, Julia Skokowa, Dominik Hartl

**Affiliations:** 1 Sir William Dunn School of Pathology, University of Oxford, Oxford, United Kingdom; 2 Center for Thrombosis and Hemostasis (CTH), University Medical Center Mainz, Mainz, Germany; 3 Institute of Cell Biology, Department of Immunology, University of Tübingen, Tübingen, Germany; 4 Department of Pediatrics I, University of Tübingen, Tübingen, Germany; 5 3rd Dept. of Medicine, Johannes Gutenberg-University Medical Center, Mainz, Germany; 6 Department of Pediatric Pneumology and Immunology, Charité Hospital, Humboldt University, Berlin, Germany; 7 Fachbereich Immunologie, Labor Berlin Charité Vivantes GmbH, Berlin, Germany; 8 Theodor Kocher Institute, University of Bern, Bern, Switzerland; 9 Sanquin Research and Landsteiner Laboratory, Academic Medical Centre, University of Amsterdam, Amsterdam, The Netherlands; 10 Department of Internal Medicine II, University of Tübingen, Tübingen, Germany; University of Basel, SWITZERLAND

## Abstract

Neutrophils, the most abundant human immune cells, are rapidly recruited to sites of infection, where they fulfill their life-saving antimicrobial functions. While traditionally regarded as short-lived phagocytes, recent findings on long-term survival, neutrophil extracellular trap (NET) formation, heterogeneity and plasticity, suppressive functions, and tissue injury have expanded our understanding of their diverse role in infection and inflammation. This review summarises our current understanding of neutrophils in host-pathogen interactions and disease involvement, illustrating the versatility and plasticity of the neutrophil, moving between host defence, immune modulation, and tissue damage.

## Neutrophil’s Life Cycle

### Granulopoiesis

Neutrophils are the predominant immune cell population in human blood, where they patrol and protect us from pathogens, and diseases with neutropenia show that they are indispensable for controlling bacterial and fungal infections. Neutrophils develop in the bone marrow from haematopoietic stem cells in a process called “granulopoiesis” and mature neutrophils are characterised by their segmented nucleus and granules that are filled with >700 proteins [1]. Bone marrow neutrophil lineage cells can be divided into three compartments: (i) the stem cell pool composed of hematopoietic stem cells and pluripotent progenitors; (ii) the mitotic pool composed of proliferating, lineage-committed myeloblasts, promyelocytes, and myelocytes; and (iii) the post-mitotic pool composed of metamyelocytes, band cells, and mature neutrophils. Post-mitotic bone marrow neutrophils constitute 95% of the neutrophils in the body [2,3] and this reserve is easily mobilized and recruited rapidly to sites of infection.

Granulocyte colony-stimulating factor (G-CSF) is the predominant factor regulating the neutrophil’s life cycle by increasing cell proliferation, survival, differentiation, and trafficking/mobilization. Mice lacking G-CSF or its receptor have a profound, but not absolute, neutropenia in bone marrow and blood [2,4,5]. However, these mice can still produce mature neutrophils in steady state and increase their production and mobilization in “emergency” situations, indicating that other signals can provide partial or complete compensation [6]. Moreover, G-CSF induces proliferation and expression of anti-apoptotic proteins and regulates chemokine expression [7,8]. However, the precise mechanisms by which G-CSF signals regulate mitotic and post-mitotic neutrophils are not fully understood. Maintenance of neutrophil numbers is further regulated by phagocytosis of apoptotic neutrophils by macrophages, a process termed “efferocytosis.” Efferocytosis reduces the production of interleukin (IL)-23 and IL-17 and dampens G-CSF production [9]. G-CSF thus regulates the neutrophil life cycle at multiple levels and, consequently, has become an important therapeutic agent for neutropenic diseases, as discussed below. Recently, autophagy has been reported as a negative regulator of neutrophil development in the bone marrow [10].

### Mobilization and trafficking

Chemokines orchestrate the balance between neutrophil release and retention. Bone marrow stromal cells produce C-X-C-motif chemokine ligand (CXCL) 12 that binds to C-X-C-motif chemokine receptor (CXCR) 4, leading to neutrophil retention, while release is mainly mediated by CXCR2 [11]. Pharmacologic CXCR2 inhibition in healthy humans, using ozone- or LPS-induced inflammation models [12–15], or in patients with severe asthma [16] or cystic fibrosis (CF) [17] showed that CXCR2 inhibition is safe and decreases neutrophilic inflammation in the airways.

Clearance of apoptotic neutrophils by macrophages, a mechanism involving liver X receptor (LXR), is essential for immune homeostasis and impaired clearance of apoptotic neutrophils has been linked to autoimmune disease [18–20]. Recent murine studies have extended this concept by highlighting the role of the bone marrow as a site of neutrophil clearance [21]. Intriguingly, homing of senescent neutrophils back to the bone marrow was found to regulate the circadian release of hematopoietic progenitors into the circulation [22]. However, the relevance of this circadian mechanism for neutrophil homeostasis in humans remains debatable. Another layer of complexity has been added by the concept of reverse neutrophil migration from peripheral organs back into the bloodstream. Reverse transmigration has been first observed in endothelial cells in vitro [23], and in mice in vivo [24], and has then been defined as a novel mechanism of inflammation resolution in zebrafish models [25]. Despite these fascinating mechanistic insights, their relevance for human diseases remains to be defined.

Released neutrophils are proposed to disseminate in the periphery into circulating and marginated neutrophil pools. The latter refers to neutrophils adherent to endothelial cells in the spleen, liver, bone-marrow, and the lung that can be recovered by exercise and adrenaline [26]. While a recent study highlights the importance of the pulmonary marginated pool in mice [27], its role in humans, at least under homeostatic conditions, remains questionable since the injection of non-primed autologous neutrophils did not lead to a significant retention in the pulmonary vasculature [28]. Further studies are required to shed more light on the marginated neutrophil pool in man and mice.

The traditional paradigm of neutrophils as short-lived immune cells has been challenged by in vivo-labelling studies, demonstrating a life span of 5.4 days for human neutrophils [29], ten times longer than previously estimated, and suggesting that neutrophils shape immune responses beyond rapid host–pathogen interactions. However, alternative explanations have been proposed [30,31] and there is no broad consensus yet regarding the life span of neutrophils in humans. Furthermore, in vivo-labelling studies are required to solve these controversies and to gain deeper insight into the neutrophil’s life span, particularly under infectious disease conditions.

### Neutrophil serine proteases, serpins, and neutrophil survival

Amongst the broad armamentarium that neutrophils carry in their granules, neutrophil serine proteases directly kill microbes and inactivate bacterial toxins [32,33]. Excessive host tissue damage and inflammation driven by the uncontrolled activity of these proteases (namely elastase, proteinase-3, and cathepsin G) is counterbalanced by endogenous serine protease inhibitors (“serpins”) [34]. Besides protecting the body from free and harmful proteolytic activities, recent studies demonstrate that serpins have an unexpectedly broader role in regulating neutrophil survival. This novel function was first hinted at by mice lacking the intracellular inhibitor Serpinb1a, which showed reduced neutrophil survival and severe bone marrow neutropenia [35], rendering these mice susceptible to bacterial and viral lung infections [36,37]. Further studies showed that Serpinb1a is critical for maintaining neutrophil survival by blocking cell-intrinsic death mediated by cathepsin G and proteinase-3 [38,39].

## Neutrophil–Pathogen Interactions

**Fig 1:**
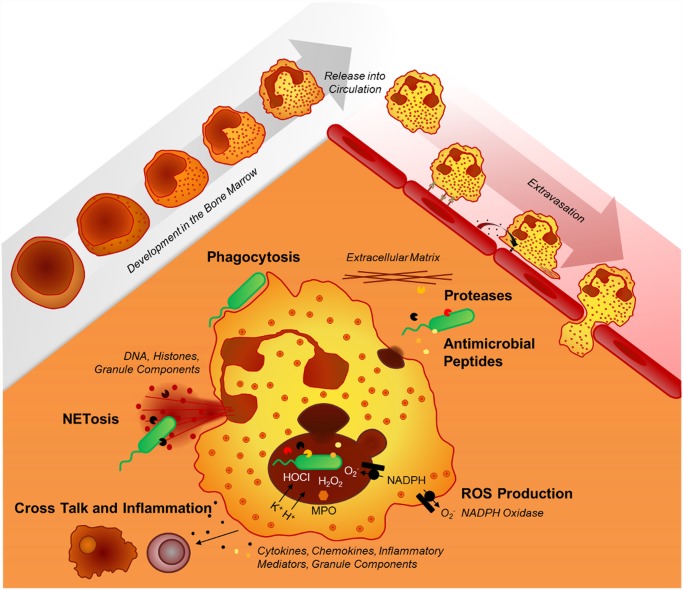
Neutrophil effector mechanisms. The mechanisms neutrophils employ to fight infections include phagocytosis, the release of various granule components into the extracellular space or into the phagosome (mainly proteases, oxidants, antimicrobial peptides), and the formation of neutrophil extracellular traps (NETs).

Recent findings have substantially expanded our view on the repertoire of antimicrobial effector functions of the neutrophil in host–pathogen interactions. Beyond phagocytosis, neutrophils employ neutrophil extracellular traps (NETs) [40] to capture microbes extracellularly and autophagy to digest them intracellularly [41,42]. Several factors decide which antibacterial effector functions are activated: (i) the presence of serum favors phagocytosis but inhibits the formation of NETs [43], (ii) transmigration triggers the release of contents from secretory vesicles and specific granules, and (iii) cell adherence lowers the threshold for integrin-dependent neutrophil activation. Importantly, intravital microscopy imaging approaches have expanded our understanding of neutrophil–pathogen interactions extensively. A recent study using this technology showed that phagocytosis and NETosis act in concert to combat *Staphylococcus aureus* in vivo, supporting a novel “neutrophil multitasking” concept in host–pathogen interactions [44]. Other in vivo studies demonstrated intracapillary neutrophil crawling in *S*. *aureus* infection [45] and dynamic and intravascular NET formation in bacterial [46] and viral infections [47]. Fig. 1 illustrates the diversity of neutrophil effector mechanisms in host–pathogen interactions.

In the paragraphs below, the key neutrophil effector functions are discussed.

### Phagocytosis

Neutrophils are rapid and potent phagocytes. Upon ligation of opsonic receptors, such as Fcγ receptors, C-type lectins or complement receptors, the neutrophil membrane engulfs a particle or a microbe, a process that is mediated by a complex interplay of membrane lipids, intracellular signalling cascades, and cytoskeletal rearrangements. Subsequently, primary and secondary granules fuse with the phagosome and discharge their antimicrobial contents. Phagocytosis occurs within minutes and is enhanced by the complement system, IgG antibodies, and cell preactivation. Once specific granules fuse with the phagosome, the nicotinamide adenine dinucleotide phosphate (NADPH) oxidase is assembled from the membrane-bound flavocytochrome *b*_558_, consisting of p91^phox^ (phagocyte oxidase) and p22^phox^ subunits, and the cytoplasmic components p47^phox^, p67^phox^, p40^phox^, and Rac. Potassium ion (K^+^) fluxes also occur in response to NADPH oxidase activation [48]. Reeves et al. hypothesised that K^+^, in turn, releases granule proteases that mediate bacterial killing, suggesting that the NADPH oxidase reaction mainly serves as an activator for proteases instead of being a microbicidal mechanism itself [49], a concept that has been challenged [50], most recently by a study showing that serine protease deficient human neutrophils show no impairment in killing bacteria [51]. The killing of *S*. *aureus* can be restored in NADPH oxidase-deficient neutrophils by adding liposomes loaded with glucose oxidase [52] or polyethylene glycol-conjugated D-amino acid oxidase [53], which produces H_2_O_2_. In these systems, neutrophils were still able to kill bacteria even when the NADPH oxidase was inactive. Moreover, inhibition of the NADPH oxidase does not impair killing of *S*. *pneumoniae* [54], and NADPH oxidase-deficient neutrophils from chronic granulomatous disease (CGD, see below) patients are still able to kill *Escherichia coli*, indicating additional microbicidal mechanisms independent of reactive oxygen species (ROS) production. A recent study further shows the involvement of the nucleotide-binding oligomerisation domain (NOD)-like receptor family, pyrin domain-containing protein (NLRP) 3 inflammasome in macrophage phagocytosis by demonstrating that activation of caspase-1 controls the phagosomal pH by regulating the NADPH oxidase (NOX) 2 to control phagosome function in gram-positive, but not gram-negative infections [55].

### Neutrophil Extracellular Traps (NETs)

**NET induction.** The concept of NETosis was introduced in 2004 [40] as a novel mechanism for neutrophils to release extracellular DNA traps, composed of decondensed chromatin, histones, and granule proteins. This finding inspired a variety of studies employing different NETosis-inducing agents, including bacteria, fungi, protozoa, viruses, platelets, nitric oxide donors, and others. NET formation has been described as a novel form of cell death [56], involving the translocation of elastase from primary granules to the nucleus, where it cleaves specific histones and leads to chromatin decondensation [57]. Other studies support the notion that even viable neutrophils can form NETs, consisting of mitochondrial [58] or nuclear DNA, in a rapid and non-lytic manner [44]. Neutrophils treated with NADPH oxidase inhibitors or cells from CGD patients show impaired NET formation in response to phorbol-12-myristate-13-acetate (PMA) or *S*. *aureus* [56], and CGD gene therapy restored NET formation [59], suggesting that ROS are indispensable for NET formation. However, other studies suggested that ROS involvement depends on the stimulus [60–63]. In addition to ROS, several other pathways have been reported to be involved in NET formation, such as peptidyl arginine deiminase (PAD) 4 [64], the Raf-MEK (mitogen-activated kinase/ERK kinase)-ERK (extracellular signal-regulated kinase) pathway [65] and autophagy [66].

**Microbial NETosis escape mechanisms**. Pathogens have evolved a variety of mechanisms to escape from host defence. One of these mechanisms is the expression of DNase to degrade NETs. *Streptococcal* DNases render the bacteria resistant to NET-mediated killing [67–69]. Similar results have been obtained with other pathogens. However, the killing capacity of NETs was questioned by a study showing that pathogens entrapped in NETs survived and retained their ability to proliferate after being released from NETs [70], an issue requiring further investigations.

**NETs in pathological conditions.** Although NET generation has been described initially as an antimicrobial mechanism, recent data suggest that NETs could play a more important role in autoimmune and autoinflammatory pathologies, such as vasculitis [71], lung injury [72,73], atherosclerosis [74], rheumatoid arthritis [75,76], thrombosis [77–80], gout [81], and systemic lupus erythematosus (SLE) [43,82]. Several strategies have been proposed to interfere with NETs, particularly digestion of NET-DNA with DNase or targeting NET-associated proteins. DNase is successfully used to treat patients with CF, and its beneficial effect may be due to cleaving NETs [83,84]. Other approaches were so far mainly restricted to animal models and require clinical studies to assess their therapeutic usefulness.

Several aspects of NET formation remain enigmatic [85]. Despite a large number of studies demonstrating NET formation in vitro, our understanding of NET formation in vivo is limited. Most studies used PMA to induce NETs, while the major driver(s) of NET formation under (patho)physiological conditions remain poorly understood. A recent study suggests that particularly larger pathogens, such as fungal hyphae, induce NET formation [86]. Another open question is why some neutrophils form NETs and others do not. This heterogeneity remains enigmatic, but recent studies indicate that NET formation could be linked to olfactomedin 4 expression by a subset of neutrophils [87]. Moreover, the biological relevance of lytic/cell death-associated versus non-lytic/viable NET formation remains an open question [88]. While viable and rapid NET formation has been established in mice, human studies are scarce, but propose a role for this rapid NET formation mechanism in *S*. *aureus* infections [60]. Neutrophils have a relatively low content of mitochondrial DNA compared to nuclear DNA. Nevertheless, mitochondrial DNA is released following physiological stimuli and may represent a distinct form of NET formation [58]. While there is evidence for an involvement of elastase, PAD-4, and histone citrullination in NET formation, the precise cellular machinery leading to NET release is incompletely defined. For example, how elastase escapes from primary granules to reach the nucleus and what physical forces lead to propulsion of nucleic acids outside the cells remain largely elusive. How important are NETs in comparison to other neutrophil effector functions with regards to bacterial killing? A recent study in Papillon-Lefèvre syndrome, where neutrophils lack active serine proteases, such as elastase, showed that patient’s neutrophils failed to form NETs, but were fully competent to kill bacteria [51]. These data indicate that serine proteases are essential for NET formation, but that NET formation, in turn, could be dispensable for killing of certain bacteria. Further studies with more bacterial, fungal, and viral pathogens are required to solve these issues.

### Antimicrobial peptides

Antimicrobial peptides play a major role at epithelial barriers and are secreted into various body fluids, such as sweat, alveolar lining fluid, milk, or intestinal mucus [89]. Most peptides in human neutrophil granules, like bactericidal/permeability-increasing protein, α-defensins, and cathelicidins, execute their microbicidal effects by disrupting bacterial membranes. Lactoferrin and lipocalin inhibit microbial iron uptake, and lysozyme cleaves cell wall peptidoglycans. Beyond their antimicrobial activities, cathelicidins (or their processed cleavage product LL-37) and α-defensins can also act as proinflammatory mediators and chemokine receptor agonists. Antimicrobial peptides, released by neutrophils, are also associated to NETs [85], facilitating a close interaction with NET-entangled pathogens.

## Neutrophils and the Microbiome

The gut microbiome has attracted high interest in various fields of research and there is now compelling evidence to support the notion that mucosal commensals regulate granulopoiesis and neutrophil homeostasis at several levels. Prolonged antibiotic treatment leads to reduced neutrophil numbers and germ-free animals present a severe neutropenia, indicating a close and homeostatic link between microbiota and neutrophils [90–92]. While our understanding of cellular and molecular mechanisms remains incomplete, the regulation of neutrophil homeostasis by the microbiome seems to be mediated by structural features of bacteria that can activate innate pattern recognition receptors, leading to increased neutrophil production [93–95]. There is also emerging evidence that metabolites, such as short chain fatty acids, produced by gut commensals from dietary fibers, regulate neutrophil-mediated inflammation through the chemoattractant receptor G protein-coupled receptor (GPR) 43 expressed at high levels by neutrophils [96]. Dysbiosis (that is, altered microbiome composition) has been associated with increased incidence and severity of various chronic inflammatory diseases and cancer [97,98], yet the interaction between microbiota and neutrophils in these conditions remains to be established. Moreover, further studies are warranted to determine whether an altered microbiome induces changes in neutrophil phenotypes and functions and, vice versa, whether targeting neutrophils has an effect on the microbiome.

## Neutrophil–Immune Cell Interactions

Neutrophils can interact with a variety of immune and non-immune cells at several levels, best studied for dendritic cells (DCs), macrophages, natural killer (NK) cells, and T cells [99,100]. Particularly, neutrophils and DCs frequently co-localize at sites of infection and DCs were found to capture neutrophils that have phagocytosed *Leishmania* and underwent apoptosis, a novel mechanism, which is how this pathogen undermines adaptive immune responses [101,102]. T helper 17 (Th17) cells orchestrate neutrophil recruitment by producing IL-17 and the neutrophil chemoattractant CXCL8. Neutrophils, in turn, recruit Th17 cells via release of the chemokines CCL20 and CCL2, resulting in a positive feedback loop between neutrophils and Th17 cells [103]. In addition, neutrophils interfere with T-cell proliferation as neutrophilic myeloid-derived suppressor cells (MDSCs). Neutrophils secrete the B-cell and plasma cell survival factors “B lymphocyte stimulator” (BLyS) [104] and “A proliferation-inducing ligand” (APRIL) [105]. In turn, a subset of splenic B cells produces GM-CSF [106] that provides differentiation signals for splenic neutrophils [107]. Human splenic neutrophils have been reported to activate marginal zone B-cell class switching and T-cell-independent antibody production, suggesting a distinct “B cell-helper neutrophil” phenotype [108]. However, these findings have recently been challenged by another study in which neutrophils with characteristics of the proposed “B cell-helper neutrophil” phenotype were not found in human spleens [109].

Murine and human studies indicate that distinct neutrophil phenotypes, initially identified as low-density neutrophils in cancer patients, inhibit T and NK cell proliferation. In analogy to monocyte subsets, those suppressive neutrophilic cells have been termed granulocytic/neutrophilic MDSCs [110]. The precise cellular mechanism(s) by which MDSCs suppress T-cell responses, however, have not been fully defined, but studies indicate that arginase, ROS, indoleamine 2,3-dioxygenase (IDO), Mac-1, PD-L1, and STAT3 might be involved [110–113]. Inter-species differences appear to exist, as mechanisms identified in mice do not seem to be always transferable to the human situation. Importantly, in systemic inflammation, a distinct subset of activated CD62L^dim^ neutrophils was found to suppress T cell proliferation mediated through a Mac-1-dependent mechanism [114].

## Neutrophil Defects

Inherited neutrophil defects exemplify the importance of this cell type for various infectious and non-infectious conditions. These defects comprise severe congenital neutropenia (SCN), cyclic neutropenia, CGD, leukocyte adhesion deficiencies (LADs), myeloperoxidase (MPO) deficiency, warts, hypogammablogulinaemia, infections, myelokathexix (WHIM) syndrome, specific granule deficiency, defects in toll-like receptor (TLR)/interleukin 1 receptor-associated kinase (IRAK) 4/caspase activation and recruitment domain-containing protein (CARD) 9 signalling, and other less defined entities and are summarized in the chapters below.

### Severe congenital neutropenia (SCN)

In SCN, neutrophilic differentiation is blocked at the promyelocyte/myelocyte stage, leading to isolated neutropenia with frequent severe bacterial and fungal infections and requiring lifelong treatment with G-CSF. Upon G-CSF treatment, SCN patients produce low numbers of neutrophils, sufficient to protect them from infections. The majority of SCN patients harbour autosomal dominant mutations in the neutrophil elastase gene (*ELANE*) [115]. Mechanistically, it has been proposed that intracellular accumulation of misfolded mutated neutrophil elastase activates the unfolded-protein response (UPR) and endoplasmatic reticulum (ER) stress [116,117] and promotes apoptosis [118]. Beyond *ELANE*, mutations in the haematopoietic cell-specific Lyn substrate (HCLS) 1-associated gene X1 (HAX1) were identified as a cause of neutropenia [119]. Further studies showed that the HAX1 interaction partner HCLS1 is activated by G-CSF, leading to the activation of the myeloid-specific transcription factor lymphoid enhancer binding factor (LEF)-1 [120,121]. A small subgroup of SCN patients have mutations in the transcriptional repressor oncoprotein growth factor independent (GFI) 1 [122], cytoskeletal regulator Wiskott-Aldrich Syndrome (WAS) protein [123], glucose-6 phosphatase catalytic subunit 3 (G6P3C) [124], endosomal adaptor protein p14 (also known as MAPBPIP) [125], or the endosomal trafficking vacuolar protein sorting 45 (VPS45) [126]. Depending on the type of the inherited gene mutation, SCN patients exhibit either isolated neutropenia (*ELANE* mutations) or additional syndromes such as lymphopenia (GFI1 gene mutations); monocytopenia (WAS-mutations); mental retardation/seizures (HAX1 mutations); prominent superficial venous network, atrial defects, and uropathy (G6PC3-associated disorders); and albinism and hyper-gammaglobulinemia (p14-mutated patients).

### Chronic granulomatous disease (CGD)

CGD is caused by mutations in the genes encoding the subunits of the phagocyte NADPH oxidase complex, with an incidence around 1/200,000 [127]. CGD phagocytes cannot effectively produce superoxide (O_2_^-^) and fail to kill ingested pathogens, leading to severe infections, mainly by *Staphylococcus* and *Aspergillus* species [127,128]. CGD disease severity correlates inversely with the remaining neutrophil ROS production capacity [129]. The majority of CGD patients show mutations in gp91^phox^, which is the only CGD gene encoded on the X-chromosome [127–131]. X-linked CGD is characterised by an early onset and a high morbidity and mortality [128,129] and can also affect female carriers with an extreme X-chromosome inactivation pattern [132]. It has been proposed that the susceptibility of CGD patients to *Aspergillus* species might be due to a defect in ROS-dependent NET formation [59], an issue requiring further research. CGD patients exhibit a state of chronic immune activation, possibly due to diminished autophagy triggering excessive IL-1β release [133]. As a result, autoimmune conditions, such as SLE, discoid lupus erythematosus, and rheumatoid arthritis, are more prevalent among CGD patients [127,128,134]. Obstructive gastrointestinal and genitourinary granulomas in CGD are usually not caused by infections but by chronic inflammation [135] and often coincide with colonic inflammation reminiscent of Crohn’s disease [127,128,136]. This may be due to diminished intestinal epithelial defence as a result of the lack of ROS production by transmigrating CGD neutrophils [137].

### Leukocyte adhesion deficiencies (LADs)

LADs are caused by several autosomal recessive genetic defects in molecules involved in the leukocyte adhesion cascade. The patients’ neutrophils cannot leave the circulation, leading to infection susceptibility [138]. In LAD-I, mutations in the gene encoding CD18, the integrin β2-chain, lead to the complete absence or decreased expression of the integrins αLβ2, αMβ2, αXβ2, and αDβ2 [139–143], rarely to normally expressed, but dysfunctional, β2-integrins [144]. Mutations in a Golgi guanosine diphosphate-fucose transporter (GFTP) protein, which is encoded by the *SLC35C1* (solute carrier family 35 member C1) gene, lead to LAD-II [145,146], which was reclassified as the congenital disorder of glycosylation-IIc (CDG-IIc) [147]. Patients with LAD-II develop milder infections as in LAD-I, but have other abnormalities [147–149]. LAD-III, also known as LAD-I/variant, is caused by mutations in the gene *FERMT3* (fermintin family homolog 3) encoding the β-integrin (β1, β2, and β3) adaptor protein kindlin-3. LAD-III patients also suffer from platelet defects and subsequent bleeding as well as osteopetrosis-like bone defects [150–154]. A combination of a LAD-like and CGD-like phenotype was described in two patients with mutations in Rac2 in which neutrophils showed defective chemotaxis due to impaired actin polymerisation and impaired NADPH oxidase activation [155,156]. An exciting, novel concept has recently emerged from studies of LAD-I patients in which defective neutrophil recruitment causes inflammatory periodontal bone loss. Surprisingly, the severe bone loss in these patients was not due to a lack of control of bacterial infections, but rather to the inability to control IL-17 production, which is normally reduced by phagocytosis of locally recruited neutrophils [157]. These findings suggest that neutrophils, the prototypic proinflammatory cells, have a profound anti-inflammatory function in steady state that is mediated by their effective efferocytosis. Thus, neutralizing IL-17 may be relevant for LAD-I and other diseases in which neutrophil recruitment to peripheral tissues is defective.

### WHIM syndrome

In WHIM syndrome, CXCR4 gain-of-function mutations lead to neutropenia caused by increased retention of neutrophils in the bone marrow [158]. In addition to neutropenia, these patients often have lymphopenia and monocytopenia. The concept of CXCR4-dependent release of neutrophils from the bone marrow has recently been challenged by an intravital microscopy study showing that CXCR4 inhibition using plerixafor in mice triggers mobilization of neutrophils from the marginated pool of the lung and simultaneously prevents neutrophil homing to the bone marrow [27], highlighting the lung as a rich and easily mobilized source of neutrophils. The therapeutic relevance of these findings for neutropenic patients and other diseases remains to be defined in future studies.

### Defects in innate immune receptor signalling

Neutrophils express various TLRs and TLR activation triggers antibacterial effector functions [159]. Mutations in myeloid differentiation primary response (*MYD) 88* or *IRAK4*, central downstream mediators of TLR signalling, lead to recurrent, invasive bacterial infections in infancy and early childhood. This susceptibility decreases with age, so TLR-mediated signalling in neutrophils seems rather dispensable beyond adolescence. Neutrophils of these patients do not respond to TLR and IL-1R ligation, whereas ROS production is normal [160,161]. Acute-phase responses are delayed or even absent despite severe infections [162–166]. Mutations in CARD9, a protein essential for the sensing of fungi, lead to *Candida* infections [167], which was recently linked to impaired neutrophil killing [168]. Mutations in proteins regulating the activity of nuclear factor (NF)κB, namely NFκB essential modulator (NEMO) and inhibitor of NFκB kinase (IKK)α, lead to immunodeficiency in combination with other symptoms [166].

### Specific Granule and MPO deficiency

Patients with specific-granule deficiency are extremely rare and suffer from recurrent bacterial infections due to a lack of secondary and tertiary granule proteins [169]. Specific-granule deficiency is caused by mutations in the transcription factor CCAAT/enhancer-binding protein ε (cEBPε), which is an important transcriptional regulator of granulopoiesis at the late stages of differentiation. Inherited MPO deficiency is frequent in humans, but MPO-deficient individuals are mostly asymptomatic, except for a small subset that suffers from diabetes [170].

## Neutrophils in Acute Inflammation

Neutrophils are the first cells recruited to sites of infection and inflammation and, therefore, play a key role in several forms of acute inflammation, a topic that goes beyond the scope of this review. Here, we highlight several recent findings in this field and refer for further information to previous, more comprehensive reviews [171–174]. Neutrophil recruitment and activation are key events in acute inflammatory conditions, such as acute lung injury, and essential to provide a first cellular shield against bacterial and fungal pathogens. Neutrophil infiltration is not limited to infectious conditions and also occurs in sterile environments, mediated by a multistep migratory sequence, involving the inflammasome, chemokines, and formyl-peptide signals [175]. Physiologically, acute neutrophilic inflammation is followed by a resolution phase [176], important for tissue homeostasis. If these resolving mechanisms fail, neutrophils drive chronic inflammation, characterized by oxidant and protease release and leading to tissue injury. Consequently, a precise understanding of the local fine-tuning of neutrophil activation at sites of inflammation is mandatory. Full-blown neutrophil activation generally requires a two-step process, initiated by preactivation/priming through proinflammatory cytokines, such as tumor necrosis factor (TNF) α, IL-1β, IL-8, leukotriene (LT)B4 or GM-CSF, or bacterial products and by a later second-hit stimulus [177]. Priming is not a terminal event, as neutrophils can undergo cycles of priming and de-priming [178]. Importantly, the lung has been demonstrated recently to be a place for retention of primed neutrophils, a protective mechanism shown to be impaired in acute respiratory distress syndrome (ARDS) [28,179]. While various stimulants of neutrophil activity have been established, negative regulators are poorly defined. A recent study demonstrates that the sialic acid binding Ig-like lectin E (siglec-E) acts as an important negative regulator of neutrophil recruitment to the lung [180]. The NADPH oxidase, well known as the major source of ROS, has paradoxically also been shown to limit inflammation [134], yet the underlying mechanisms remained poorly defined. Davidson and coworkers now show that the NADPH oxidase dampened neutrophilic inflammation and was protective against acute lung injury by activating nuclear factor erythroid 2-related factor 2 (Nrf2), a transcriptional factor inducing antioxidative and cytoprotective pathways and suggesting Nrf2 as a novel anti-inflammatory therapeutic target [181]. Other recently described mechanisms in acute neutrophilic inflammation include the pulmonary endothelial glycocalyx [182] and myeloid-related protein-14 [183] in sepsis, midkine [184], hematopoietic progenitor kinase 1 (HPK1) [185], and chitinase-like proteins [186].

## Neutrophils in Chronic Disease and Tissue Injury

While the role of neutrophils in acute inflammation is rather well established, their complexity in chronic diseases, tissue injury, and repair has just begun to evolve. Several chronic inflammatory disease conditions are characterized by a sustained influx of neutrophils, such as CF, chronic obstructive pulmonary disease, rheumatoid arthritis, nephritis, SLE, and cardiovascular diseases. Chemokines, lipid mediators, complement fragments, and tissue breakdown products trigger the migration of neutrophils into diseased tissues. Upon local activation, transmigrated neutrophils release their toxic ingredients, proteases and oxidants, which cause tissue injury and drive the production of chemokines that feed into the neutrophil recruitment loop. Neutrophil-derived serine and matrix metalloproteases (MMPs) cleave extracellular matrix (ECM; elastin and collagen) and impair host defence by degrading immune receptors and collectins. Neutrophils are a prominent source of MMPs, particularly MMP-9, thereby contributing to tissue damage and remodelling [187]. Consequently, neutrophil activities lead to cleavage of ECM into small fragments that, in turn, can stimulate the immune system and feed a positive feedback loop of tissue destruction and immune cell infiltration. Central to tissue injury and repair circuits is the tripeptide proline–glycine–proline (PGP), which is generated through the concerted action of several neutrophil-derived proteases, particularly MMP-8, MMP-9, and prolyl endopeptidase (PE). PGP can act as a chemokine mimetic and triggers neutrophil migration through CXCR1 and CXCR2 [188]. PGP is inactivated through a non-canonical functionality of the enzyme leukotriene A4 hydrolase (LTA4H) [189]. Persistence of PGP has been found in several neutrophilic diseases, including CF lung disease. Another neutrophil-derived protein involved in inflammation and tissue remodeling is high mobility group box protein-1 (HMGB1), a chromatin protein released from necrotic neutrophils and activating receptor for advanced glycation endproducts (RAGE) TLR and CXCR4 [190,191].

## Neutrophil Heterogeneity and Plasticity

Heterogeneity of neutrophils is found at several levels: (i) nuclear appearance (band cells, mature and hypersegmented neutrophils); (ii) density (“low”-MDSCs versus “high” conventional density neutrophils [192]); (iii) NET-releasing versus non-NET-releasing neutrophils; and (iv) receptor expression profiles associated with distinct neutrophil subsets, which are summarized below:

- Olfactomedin 4-expressing neutrophils, associated with a NET-releasing neutrophil subtype [87]
- CD177 (NB1)-expressing neutrophils, associated with proteinase 3 and autoimmune diseases [193]
- CD16^bright^CD62L^dim^ for suppressive neutrophils in systemic inflammation [114]
- CD66b/CD33-expressing low-density neutrophils for neutrophilic MDSCs [192]
- CXCR4^+^ for aged/senescent neutrophils [194]
- CD63-expressing neutrophils in the airways of patients with CF [195]
- CD49d-expressing neutrophils in atopic individuals [196]
- VEGF-induced MMP-9 expressing “pro-angiogenic” neutrophils [187]
- Neutrophils expressing a T-cell receptor-associated variable immunoreceptor complex [197]
- ICAM-1/CD54-expressing neutrophils associated with systemic inflammation and reverse migration [23,198]
- N1/N2 tumor-associated neutrophils (TANs) in murine tumor models (antitumorigenic, proinflammatory N1 neutrophils; protumorigenic, immunosuppressive N2 neutrophils) [199]
- PMN-I (producing IL-12 and CCL3, inducing classically-activated macrophages) conferring protection against methicillin-resistant *S*. *aureus* (MRSA) infection and PMN-II (producing IL-10 and CCL2, inducing alternatively-activated macrophages) conferring susceptibility for MRSA infection [200]

The disease associations and functional characteristics of these described neutrophil phenotypes remain to be established. Could these markers be useful for subtyping different harmful versus beneficial neutrophil subsets at sites of chronic inflammation? Further studies in humans and mouse models are required to link phenotypic characteristics with functions.

Beyond heterogeneity, the potential of neutrophils to undergo phenotypic and/or functional plasticity has been addressed by some studies, demonstrating that neutrophils can transdifferentiate into: (i) DC-neutrophil hybrids upon GM-CSF stimulation with phenotypic and functional characteristics of both neutrophils and DCs [201,202]; (ii) DC-like cells in rheumatoid arthritis [203]; (iii) MHC-II-expressing antigen-presenting cells, induced by acidosis, cytokines, and growth factors and found in chronic inflammatory diseases [204,205]; and (iv) giant phagocytes, associated with autophagy [206]. Moreover, under inflammatory conditions, band-stage neutrophils were recently shown to transdifferentiate into monocytes [207]. Metabolic reprogramming, including the induction of anabolic pro-survival pathways, has been described in CF airway neutrophils, including up-regulation of the mammalian target of rapamycin (mTOR) pathway [208] and nutrient/glucose transporters [209]. Monocytic MDSCs have been shown to transdifferentiate into neutrophilic MDSCs [210]. While some of these neutrophil transdifferentiation phenotypes are found in chronic inflammatory and infective disease conditions [100,203,208], their overall disease relevance remains to be better defined.

## Conclusions

Novel technologies, such as intravital microscopy and transgenic mice, have expanded our insights into neutrophil homeostasis and effector functions tremendously. Neutrophil steady-state homeostasis is not only regulated by growth factors, cytokines, and chemokines, but also involves microbial pattern recognition, linking hematopoiesis with microbiota. Neutrophil clearance seems to be even more complex, involving apoptosis/efferocytosis, reverse migration, and homing of senescent neutrophils back to the bone marrow, a process that, in turn, may regulate circadian neutrophil turnover. Besides their role as potent phagocytes, neutrophils expose their granule contents and DNA through NETosis, but its precise pathophysiological relevance in vivo remains to be defined. The spectrum of neutrophil plasticities has been further expanded by their role as immunomodulatory MDSCs that dampen T-cell activity. The clinical importance of neutrophils in host defence is underscored by the severity and complexity of infections in patients with inherited immunodeficiencies associated with a loss of neutrophil number or function. Faced with the emerging heterogeneity and plasticity of neutrophils, the challenge for the future remains how to target harmful, while promoting protective, neutrophil phenotypes. Based on these concepts, neutrophils in chronic inflammatory disease conditions are supposed to do more harm than good, rendering them promising therapeutic targets in chronic diseases. However, targeting neutrophils always bears the risk of increasing infection susceptibility, particularly towards bacterial and fungal pathogens. Small-molecule CXCR2 antagonists inhibit neutrophil chemotaxis and have been used in clinical studies in CF lung disease, with limited success [211]. Beyond these universal targeting approaches, inhibiting or depleting specific harmful neutrophil subsets, while preserving beneficial subtypes, would be preferential. However, this approach is limited by our current understanding of neutrophil heterogeneity. Which markers could be useful to identify harmful neutrophil phenotypes? Future studies using small molecules or antibodies targeting distinct neutrophil subsets will be required to address this question and to move forward towards a neutrophil subtype-selective pharmacologic treatment strategy.
